# Experimental validation of a voxel-based finite element model simulating femoroplasty of lytic lesions in the proximal femur

**DOI:** 10.1038/s41598-022-11667-x

**Published:** 2022-05-09

**Authors:** Amelie Sas, An Sermon, G. Harry van Lenthe

**Affiliations:** 1 Biomechanics Section, Department of Mechanical Engineering, KU Leuven, Celestijnenlaan 300C, 3001 Leuven, Belgium; 2 Department of Traumatology, University Hospitals Gasthuisberg, Leuven, Belgium; 3 Department of Development and Regeneration, KU Leuven, Leuven, Belgium

**Keywords:** Biomedical engineering, Bone metastases

## Abstract

Femoroplasty is a procedure where bone cement is injected percutaneously into a weakened proximal femur. Uncertainty exists whether femoroplasty provides sufficient mechanical strengthening to prevent fractures in patients with femoral bone metastases. Finite element models are promising tools to evaluate the mechanical effectiveness of femoroplasty, but a thorough validation is required. This study validated a voxel-based finite element model against experimental data from eight pairs of human cadaver femurs with artificial metastatic lesions. One femur from each pair was left untreated, while the contralateral femur was augmented with bone cement. Finite element models accurately predicted the femoral strength in the defect (R^2^ = 0.96) and augmented (R^2^ = 0.93) femurs. The modelled surface strain distributions showed a good qualitative match with results from digital image correlation; yet, quantitatively, only moderate correlation coefficients were found for the defect (mean R^2^ = 0.78) and augmented (mean R^2^ = 0.76) femurs. This was attributed to the presence of vessel holes in the femurs and the jagged surface representation of our voxel-based models. Despite some inaccuracies in the surface measurements, the FE models accurately predicted the global bone strength and qualitative deformation behavior, both before and after femoroplasty. Hence, they can offer a useful biomechanical tool to assist clinicians in assessing the need for prophylactic augmentation in patients with metastatic bone disease, as well as in identifying suitable patients for femoroplasty.

## Introduction

Metastatic bone disease patients have an increased risk for pathological fractures^1,2^. If a pathological fracture occurs in a weight-bearing bone such as the femur, this leads to a severe decrease in quality of life and an increase in mortality^2–4^. Prevention of such fractures is therefore of major interest. Prophylactic surgery is advised for patients with a high fracture risk and typically involves mechanical stabilization with a prosthesis or osteosynthesis. An alternative and less invasive procedure is femoroplasty, where bone cement is injected percutaneously into the weakened proximal femur^5–7^. If the proximal femur could be safely repaired using femoroplasty instead of conventional surgical fixation, the patient would benefit from a shorter and less invasive surgical procedure, reduced recovery time, and a shorter hospital stay, all at a lower cost^6,8^. Femoroplasty has already proven to effectively relieve pain and improve functionality, yet some reports have indicated a substantial number of post-operative fractures^6,9^. As one of the long weight-bearing bones, the femur is vulnerable to fracture and uncertainty remains whether femoroplasty alone can provide sufficient mechanical strengthening^10^.

Subject-specific finite element (FE) models based on computed tomography (CT) scans are promising tools to evaluate the mechanical effectiveness of femoroplasty. They have shown to be strong predictors of the risk for fracture in patients with bone metastases^11–14^. By extending these models to simulate femoroplasty, they could also predict the risk for fracture after treatment and thereby aid in selecting appropriate patients for whom femoroplasty provides sufficient increase in strength^8,10^. Moreover, FE models are efficient for analyzing large datasets and could therefore be used to explore the effectiveness of femoroplasty on a large number of cases^10,15^. However, validation studies are required first to demonstrate the accuracy and precision of FE models simulating femoroplasty.

Only few studies have been published on the validation of FE models simulating femoroplasty^15,16^. In these studies, the models have been validated by comparing the stiffness and strength as determined by FE against the stiffness and strength determined from in vitro mechanical testing on cadaveric femurs. Given that metastatic lesions cause local effects on the displacement and strain distribution^17^, a validation of the surface deformations would offer an added value by providing further insights into the mechanical consequences of the lesions. Typically, surface strains have been measured with strain gauges attached to the cortex, but these data are limited to a small number of measurements at pre-selected spots^18^. A better spatial resolution can be obtained with digital image correlation (DIC), a non-contact optical method that can measure the full-field displacement and strain distribution over the surface of an object. The use of DIC for the validation of FE models of the femur is relatively new, explored for the first time in 2011 on composite bones^19^ and more recently on human cadaveric femurs^20–24^. DIC enables to validate the surface deformations over an extensive region, hence offering a more comprehensive validation going beyond overall measures as stiffness and strength.

Therefore, the goal of the present study was to validate an FE model for the simulation of femoroplasty in metastatic lesions, both in terms of global strength and local displacement and strain measurements from DIC. For this validation, we made use of experimental results from a previous study on eight pairs of cadaver femurs with artificial metastatic lesions^17^.

## Methods

### Mechanical experiment

Eight pairs of fresh-frozen human cadaveric femurs with artificial lytic lesions have been mechanically tested until failure (Table 1). Ethical approval was granted by the Ethics Committee of the University Hospitals Leuven (reference number NH019 2018-09-02). The specimens were obtained from voluntary donors who provided written informed consent during their lifetime to the use of their bodies for research and education. All study procedures were conducted in accordance with the guidelines approved by the Ethics Committee.

**Table 1:** Subject data.

Lesion type			Sex	Age (years)	Augmented femur
Neck	Medial	(N–M)	F	82	Right
	Anterior	(N–A)	F	60	Right
	Superior	(N–S)	M	87	Left
	Posterior	(N–P)	F	84	Left
Inter-troch	Medial	(I–M)	M	70	Right
	Anterior	(I–A)	M	62	Right
	Lateral	(I–L)	F	63	Left
	Posterior	(I–P)	F	68	Left

**Figure 1:**
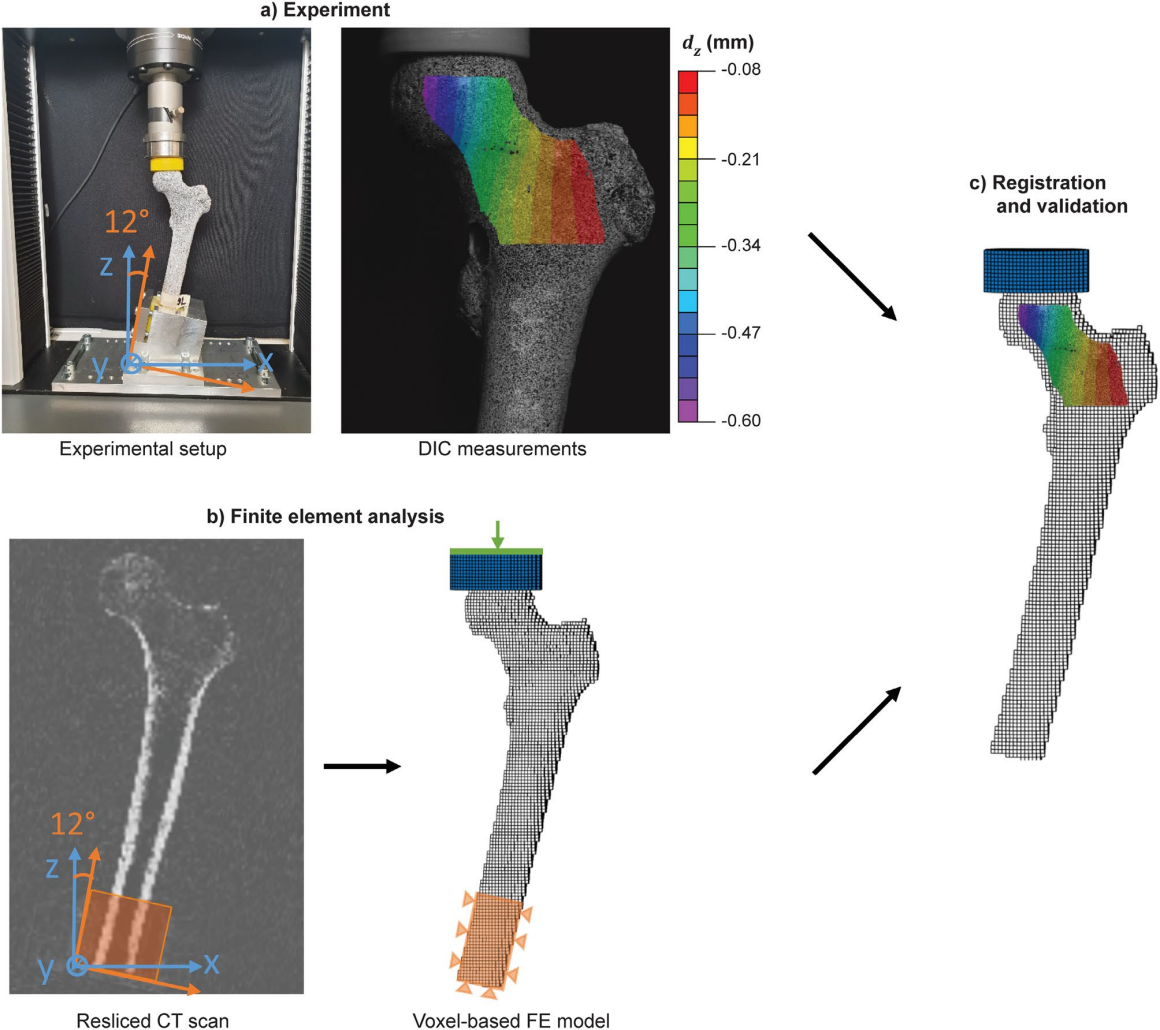
Overview of the validation workflow. (**a**) Cadaver femurs were mechanically tested in a single leg stance configuration and displacements and strains were measured using digital image correlation (DIC). (**b**) CT scans of the femurs were resliced along the experimental coordinate system (blue) and downscaled to a 2 mm voxel size to enable direct conversion into a voxel-based finite element (FE) model mimicking the experimental conditions. (**c**) The FE models were validated by comparing the strength against the experimental failure load and the surface displacements and strains against the registered DIC measurements.

The experimental protocol has been reported in detail in a previous study^17^. In short, the femurs were cut at a length of 25 cm as measured from the top of the head and stripped of soft tissues. Identical defects were milled in the left and right femur of each pair to simulate metastatic lytic lesions. Four pairs received a spherical lesion in the neck and the other four an ellipsoidal lesion in the intertrochanteric region, each at the medial, superio-lateral, anterior and posterior side respectively. One femur of each pair was left untreated (defect group), while the contralateral femur was augmented with polymethylmethacrylate (PMMA, 5–10 ml) bone cement (augmented group). Each femur was CT scanned in a water basin on top of a K_2_HPO_4_ (KHP) calibration phantom (Model 3 CT phantom, Mindways Software Inc., Austin, TX, USA) for converting the Hounsfield units to equivalent bone density (${\rho }_{KHP}$). The following scanner settings were applied (Siemens Somatom Force, Siemens AG, Germany): 120 ref kV, 250 ref mAs, slice thickness 0.4 mm, slice increment 0.2 mm, pitch 0.85, in-plane resolution 0.4 mm and bone kernel. CT scans were made at three different time points: before creation of the lesion (intact scan), after creation of the lesion (defect scan), and, if applicable, after augmentation (augmented scan)*.* For registration purposes, four tantalum markers (0.8 mm) were inserted in each femur before scanning. The femurs were mechanically tested in single leg stance configuration until failure. An increasing force (10 N/s) was applied through a PMMA cup that was placed between the loading plate and the femoral head. The distal ends of the femurs were embedded in PMMA and clamped with the shaft under an angle of 12° with the loading axis (Fig. 1a). Prior to mechanical testing, a speckle pattern was applied at the anterior surface of the femur to allow for DIC measurements. Two cameras (Grasshopper3, Flir Systems Inc., 5 Mpx) recorded the surface of the femur at a frequency of 5 Hz.

Vic-3D software (Correlated Solutions Inc., Irmo, SC) was used to perform DIC on the speckle images to retrieve the displacements and strains at the bone surface. A region of interest was drawn on the images covering the proximal part of the femur (head, neck and intertrochanteric region) (Fig. 1a). Correlation was performed using a subset size of 29 px and a step size of 6 px^17^. Thresholds were applied to remove low quality datapoints from the measurements (consistency threshold 0.02 px, confidence margin of 0.05 px and epipolar threshold of 0.5 px). In addition, the five outermost layers of datapoints were removed as these are more prone to artefacts due to the higher surface curvature and smaller search region. Engineering strains were then calculated from the displacement vectors and smoothed using a spatial decay filter (90% center-weighted Gaussian) of 11 px.

### Finite element analysis

#### Segmentation and registration of CT data

**Figure 2:**
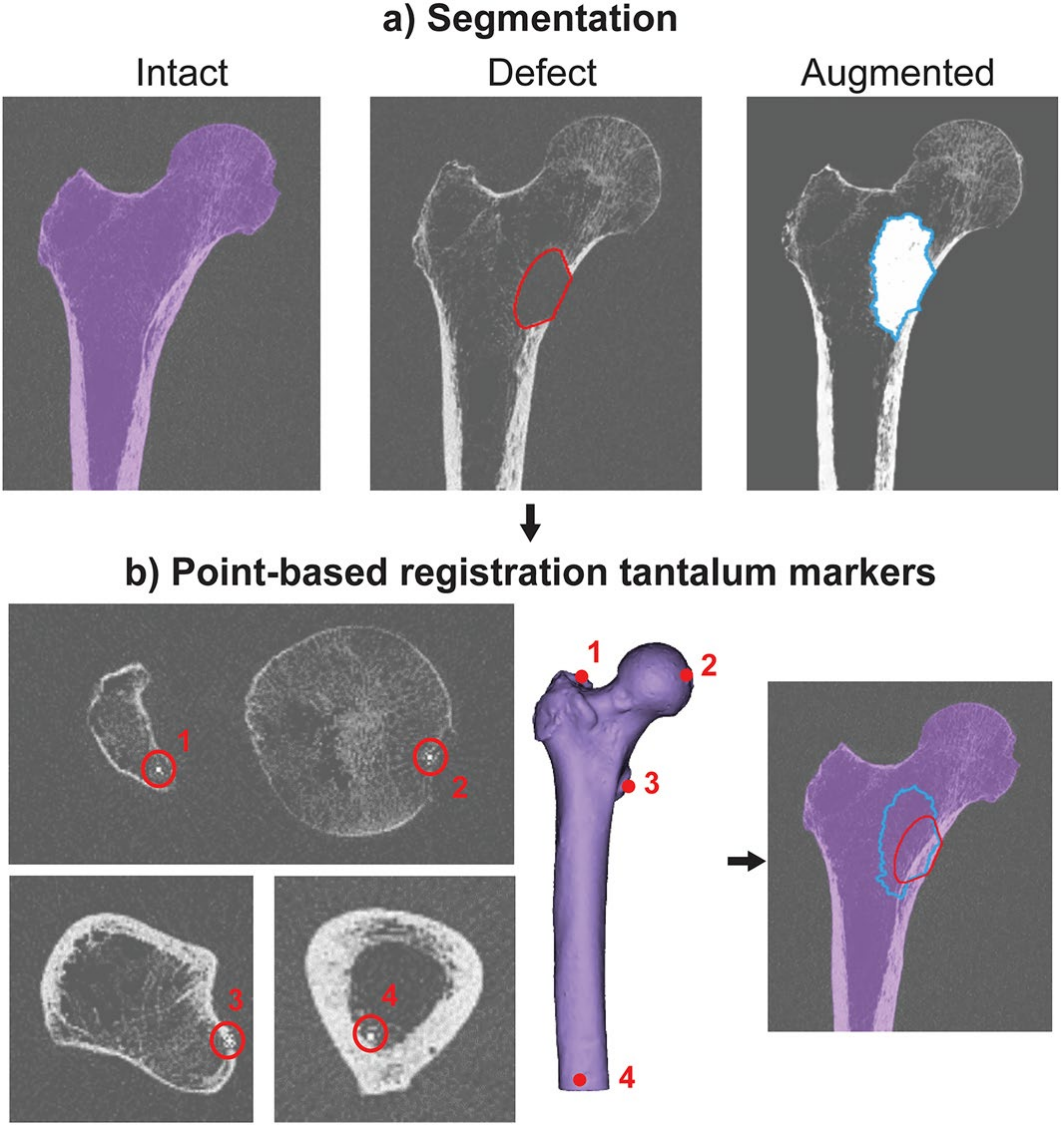
Segmentation and registration. (**a**) The femur, lesion and cement contour were segmented from the intact, defect and augmented CT scan respectively. (**b**) The lesion and cement contour were superimposed on the intact CT scan after a point-based registration of the four tantalum markers in each femur [at the top of the greater trochanter (1), the fovea of the femoral head (2), the lesser trochanter (3) and the posterior endosteal surface at the distal end of the femoral shaft (4)].

The femur geometry was semi-automatically segmented from the intact CT scans (threshold, dilation and erosion, manual correction) in Mimics 22.0 (Mimics Innovation Suite, Materialise NV, Haasrode, Belgium). The contour of the lesion and bone cement were segmented from the defect and augmented scans respectively. Both contours were superimposed on the intact scan after a rigid registration based on the position of the four tantalum markers in the CT scans (Fig. 2)^17^. The registration allowed to use a single CT scan to create FE models from the intact as well as the defect and augmented femur. For the latter two cases, the contours of the lesion and cement were converted into masks, to which different material properties could be assigned. Additionally, the geometry of the PMMA embedding of the femoral shaft was segmented from the CT scan as a reference for the experimental coordinate system. The latter was aligned with the edges of the embedding, except for a rotation of 12° along the experimental y-axis (Fig. 1a).

#### Meshing and boundary conditions

To mimic the experimental setup, a rigid transformation was performed to align the image axes of the intact CT scan with the experimental coordinate system. The CT scan was resliced in Mimics along the new coordinate axes and the voxel size was downscaled to 2 mm sized cubical voxels (Fig. 1b), the same resolution as in our previously validated FE model for fracture risk assessment^25^. Before reslicing, any negative ${\rho }_{KHP}$ values were set to 0 g/cm^3^ to decrease the effect of residual air bubbles. The resliced CT scans were converted into a hexahedral mesh using an in‐house routine that prepares the required mesh format for the FE solver ParOSol^25^. Boundary conditions mimicking the experimental setup were applied (Fig. 1b). A loading cup was added at the proximal end with a diameter of 50 mm and a concave end that was bonded to the femur head. A displacement was applied along the experimental z-axis through a stiff plate on top of the loading cup. Displacements along the x- and y-axis and rotational movements were unconstrained. At the distal end, the embedded part of the femur shaft was fully fixed.

#### Material properties and simulation

The loading cup and plate were assumed isotropic with a Young’s modulus of 2 GPa and 200 GPa, respectively. To model the defect femur, all elements corresponding to the lesion mask were removed from the FE model. To model the augmented femur, elements corresponding to the cement mask were assigned an isotropic, elastic perfectly plastic behavior with material constants as determined previously^26^. Specifically, cement within the lesion was assigned a Young’s modulus of 5 GPa and an ultimate strength of 85.9 MPa, while the bone-cement composite in the surrounding trabecular bone received a Young’s modulus of 5.7 GPa and an ultimate strength of 77.9 MPa.

For the remaining elements of the femur, identical material relationships were applied as in a previous study^25^, where we modeled the nonlinear isotropic material behavior as proposed by Keyak et al.^27^. Details on the applied material relationships are provided in Supplementary Material (S1). Since the previous study defined the material properties in function of hydroxyapatite (HA) equivalent bone density (${\rho }_{CHA}$), the following conversion was applied to the ${\rho }_{KHP}$ values from the CT scan^28^: ${\rho }_{CHA}$ = 1.15 ${\rho }_{KHP}$ – 0.0073 (g/cm^3^). Similar to our previous study, a thresholding and smoothing operation was performed to account for the partial volume effect present in voxel-based meshes^25^. Subsequently, the ${\rho }_{HA}$ values were converted to ash density (${\rho }_{ash}$ = 0.887 ${\rho }_{CHA}$+ 0.0633)^27^. Nonlinear material properties, including elasticity, a von Mises yield criterion and strain softening^27^, were assigned on an element-by-element basis as a function of the ${\rho }_{ash}$ values. Increased strength properties were applied to the surface elements of the femur head that were directly connected to the loading cup to prevent severe distortion^25^. These elements were assigned a Young’s modulus of 20 GPa and an ultimate strength of 200 MPa. All materials in the model received a Poisson ratio of 0.3. The models were solved using the open-source software ParOSol^29^, a dedicated voxel-based FE solver, combined with a previously implemented routine to account for non-linear material properties^25^.

### Validation

The FE model was validated by evaluating the accuracy in quantifying strength as well as surface displacements and strains.

Strength accuracy was evaluated by comparing the FE predicted failure load (${F}_{FE}$) with the experimentally measured failure load (${F}_{exp}$). ${F}_{FE}$ was defined as the maximal reaction force at the top of the loading cup. ${F}_{exp}$ was the maximal force recorded by the load cell during mechanical testing. The relationship between ${F}_{FE}$ and ${F}_{exp}$ was computed with linear regression analysis, and the coefficient of determination (R^2^) and the root mean squared error (RSME) were calculated. The prediction error was calculated for each specimen as ${F}_{FE}- {F}_{exp}$ and the mean and standard deviation over all specimens were quantified.

To compare the surface displacements and strains in the FE models against the DIC measurements, the DIC datapoints were registered over the surface of the FE model using an iterative closest point approach (Fig. 1c). For every DIC point, the FE nodes and elements within a distance of 4 mm were identified. The FE displacement and strain results were interpolated over these nodes and elements respectively using an inverse distance-weighted interpolation. The interpolated FE results were compared against the DIC measurements at an identical force equal to 75% of the maximal experimental force. The displacement and nominal strain magnitude along the loading axis were analyzed separately through an ordinary and robust linear regression analysis respectively. The slope and the intercept of the regression line were determined, as well as the R^2^ and the normalized root mean squared error (NRMSE). The NRMSE was computed by dividing the RMSE by the min–max range*.*

## Results

### Validation of bone strength

**Figure 3:**
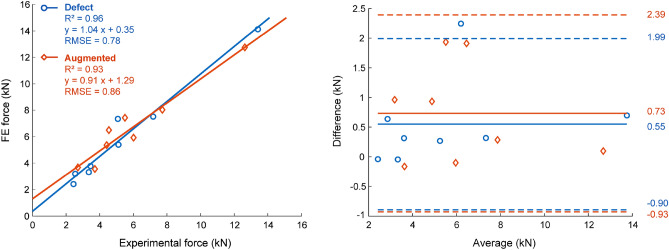
Validation failure force. A strong linear relation was found between the failure force quantified by FE and the experimental failure force, both for the defect and augmented group. Correlation graphs (left) and Bland–Altmann plots (right) are depicted.

The failure load quantified by the FE model corresponded very well with the experimental failure load, both for the defect and augmented group. In the defect group, a strong linear relation (R^2^ = 0.964, p < 0.001, RMSE = 0.78 kN) was found between ${F}_{FE}$ and ${F}_{exp}$ (Fig. 3) with a slope not different from one and an intercept not different from zero. The prediction error indicated that the failure load was overestimated by an average of 0.55 kN (± 0.74 kN). Analogously, in the augmented group, a strong linear relation was found between ${F}_{FE}$ and ${F}_{exp}$ (R^2^ = 0.926, p < 0.001, RMSE = 0.86 kN) with again a slope not different from one and an intercept not different from zero. The FE models overestimated the strength of the augmented femurs by an average of 0.73 kN (± 0.85 kN). Force–displacement curves for all specimens are provided in Supplementary Material (S2).

### Validation of surface displacements and strains

**Figure 4:**
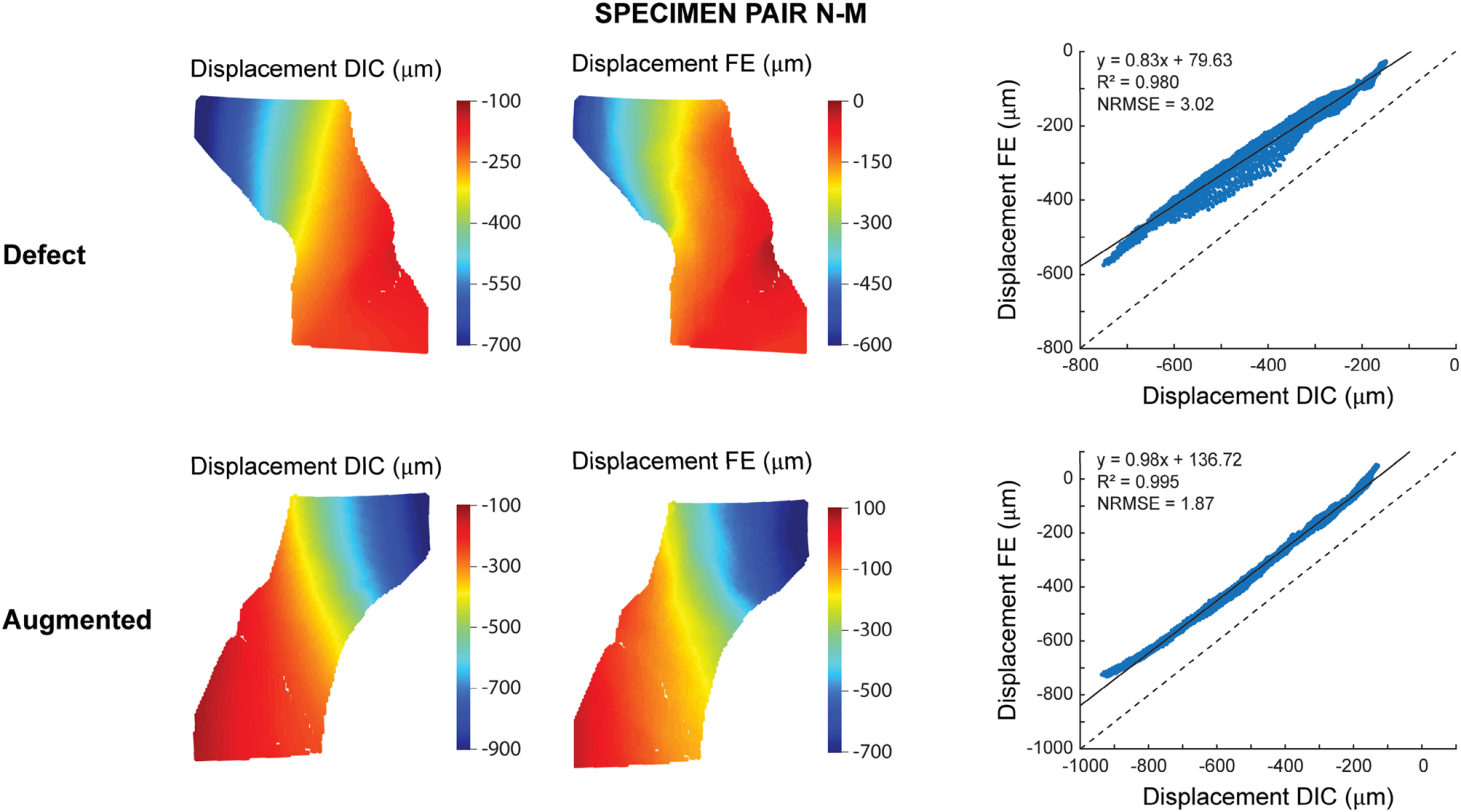
Validation displacements. (Left) Qualitative comparison of the DIC and FE displacements at the anterior surface of the proximal femur. The results are displayed for one representative specimen pair (N–M) at 75% peak experimental force. Note that the scales for the FE and DIC measurements were not matched to allow a better visual comparison of the relative distribution. (Right) Quantitative comparison of the displacement values through linear regression analysis.

**Figure 5:**
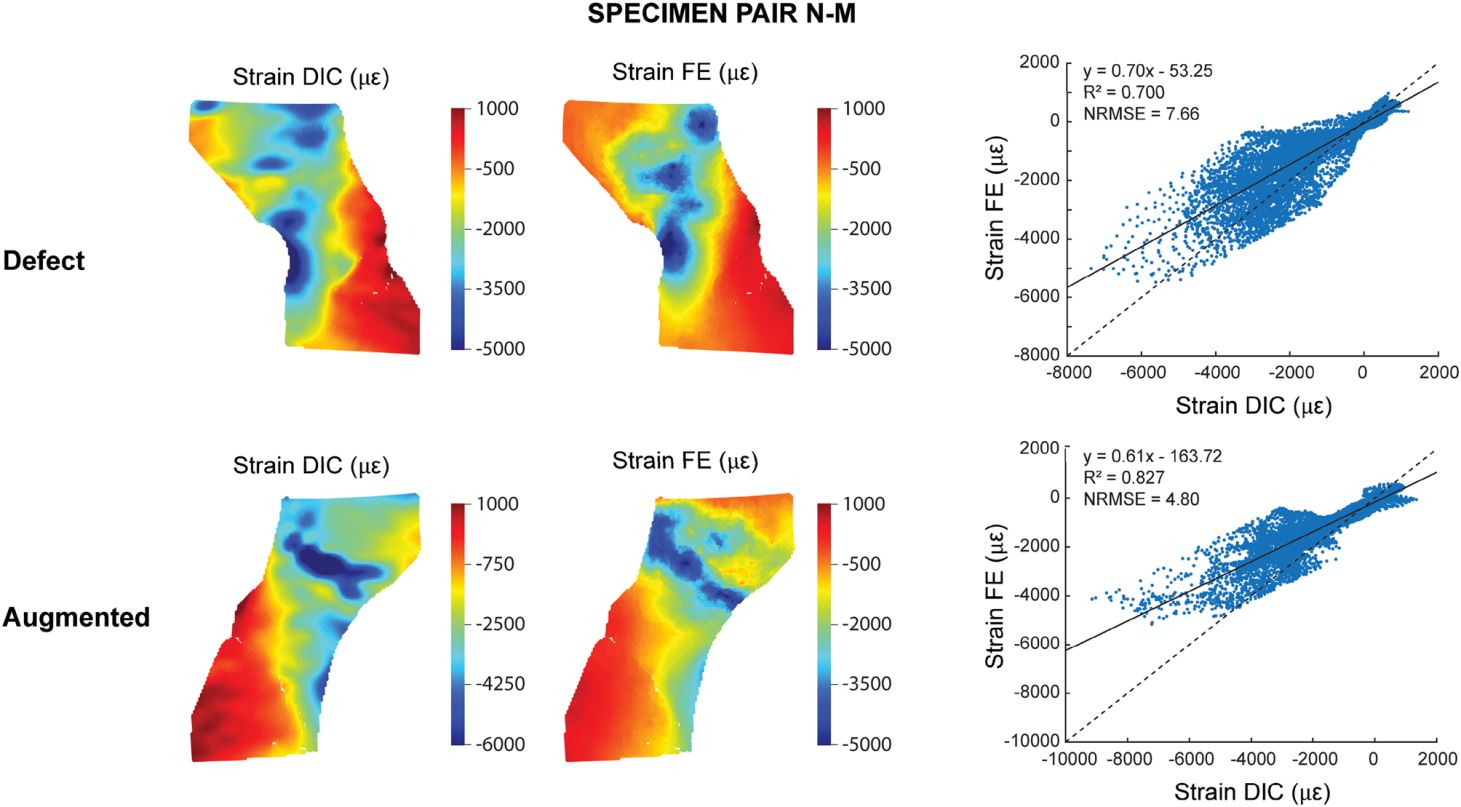
Validation strains. (Left) Qualitative comparison of the nominal DIC and FE strains at the anterior surface of the proximal femur. The results are displayed for a representative specimen pair (N–M) at 75% peak experimental force. Note that the scales for the FE and DIC measurements were not matched to allow a better visual comparison of the relative distribution. (Right) Quantitative comparison of the strain values through linear regression analysis.

Two femur pairs (N–A and N–P) were excluded from the DIC validation. N–A was excluded due to the presence of numerous holes on the anterior cortex, which largely affected the DIC results during mechanical loading (loosening of paint, leakage of marrow). N–P was excluded due to an experimental error during camera recording. For all other specimens, including defect and augmented femurs (N = 12), the surface displacements and strains from FE and DIC on the anterior surface were both qualitatively and quantitatively compared. For one representative specimen pair (N–M) the displacements (Fig. 4) and strains (Fig. 5) are included in this paper; figures for the remaining specimens are provided in Supplementary Materials (S3–S4).

A strong linear agreement was found between the DIC and FE displacements (Fig. 4). All correlations had an R^2^ > 0.98 and a NRMSE < 5%, apart from two cases with an R^2^ = 0.95 and 0.88, respectively (Table 2). However, the intercept was consistently larger than 0 (145 µm < intercept < 597 µm) and the slope was mostly close to 1, but showed some variations from 0.55 up to 1.50 (Table 2).

**Table 2:** Summary of the linear regression results for the displacement and nominal strain comparisons between FE and DIC results.

	Displacement								Nominal strain							
	Defect				Augmented				Defect				Augmented			
	R^2^	NRMSE (%)	a	b (µm)	R^2^	NRMSE (%)	a	b (µm)	R^2^	NRMSE (%)	a	b (µε)	R^2^	NRMSE (%)	a	b (µε)
N–M	0.980	3.02	0.83	80	0.995	1.87	0.98	137	0.700	7.66	0.70	− 53	0.827	4.80	0.61	− 164
N–S	0.999	1.13	1.27	256	0.999	1.12	1.24	232	0.833	7.08	0.81	− 19	0.813	8.75	0.87	− 58
I–M	0.984	4.93	1.50	448	0.951	5.04	0.87	361	0.749	9.60	0.75	351	0.653	7.78	0.56	603
I–A	0.993	2.21	1.03	474	0.985	2.57	0.85	458	0.904	3.89	0.68	67	0.797	4.74	0.5	163
I–L	0.998	1.50	1.45	597	0.995	1.96	1.06	421	0.776	10.38	1.02	-43	0.861	5.04	0.66	105
I–P	0.877	4.95	0.55	145	0.982	3.73	1.10	397	0.736	7.22	0.55	643	0.629	7.78	0.51	479
Mean	0.972	2.96	1.11	333	0.985	2.71	1.02	334	0.783	7.64	0.75	158	0.763	6.48	0.62	188
SD	0.047	1.67	0.37	204	0.018	1.43	0.15	124	0.074	2.28	0.16	282	0.097	1.81	0.14	300

a = slope; b = intercept.

The abbreviations on the left refer to the location of the lesion in the tested femurs (see Table 1).

**Figure 6:**
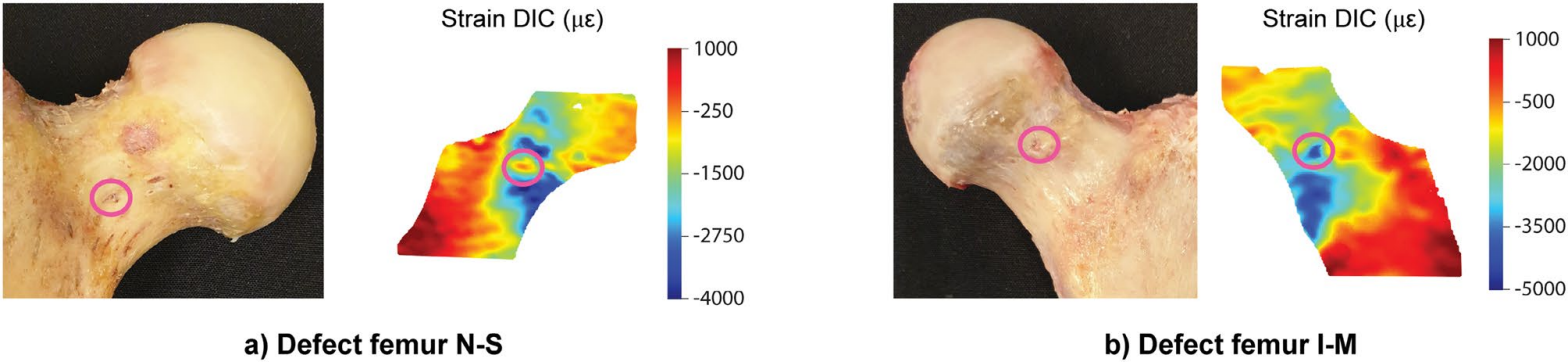
Local strain concentrations at vessel holes. Two examples [defect femurs from pair N–S (**a**) and pair I–M (**b**)] illustrate how vessel holes can result in local strain concentrations in the DIC measurements.

For the nominal strains, also significant correlations were found between the FE and DIC results with a mean R^2^ of 0.78 and 0.76 and a mean NRMSE of 7.64 and 6.48% for the defect and augmented femurs respectively (Fig. 5, Table 2). The intercept was close to 0, but the slope was typically lower than 1 (Table 2). Qualitatively, a good match was observed between the relative strain distributions in all specimens, except for some local discrepancies at the locations of vessel holes. These holes resulted in local strain concentrations in the DIC measurements (Fig. 6), which were not observed in the FE results.

Both for the displacement and strain validation, no clear differences in results were observed between the defect and augmented femurs, except for a slightly smaller slope in the augmented group.

## Discussion

The goal of this study was to validate FE models simulating femoroplasty of metastatic lesions in the proximal femur against results from mechanical testing. In contrast to previous validation studies^15,16^, we did not only validate the FE model in terms of global strength prediction, but also compared the local displacement and strain distributions on the specimen surface against measurements using DIC.

The voxel-based model accurately predicted the strength before and after cement augmentation (R^2^ = 0.96 and 0.93, RMSE = 0.78 and 0.86 kN respectively). This R^2^ value is higher in comparison to two other studies that have reported previously on the FE validation of simulating femoroplasty^15,16^. In the first study, Kaneko et al. reported an R^2^ of 0.77 and 0.88 for predicting the experimental strength of intact femurs and femurs after augmentation of artificially created lesions, respectively^15^. In their study, they used voxel-based FE models similar to ours, yet with a larger element size (3 mm versus 2 mm) and different material properties for the cement augmented region. Besides these modeling differences, the discrepancy in performance might also be related to the differing datasets; Kaneko et al. used a larger number of femurs (12 pairs), most of which came from donors who died from cancer. A second study also validated an FE model for simulating femoroplasty (9 pairs), be it in a slightly different context, i.e. for treatment of osteoporosis^16^. They found a linear relationship between the experimental and FE predicted yield force with an R^2^ of 0.87 and 0.72 before and after augmentation of osteoporotic femurs, respectively.

A strong correspondence was found between the FE and DIC displacements on the anterior surface, both for the defect and augmented femurs. All correlations had an R^2^ > 0.98 and a NRMSE < 5%, apart from two cases with an R^2^ of 0.95 and 0.88. Despite the strong correlations, the slope and intercept of the observed relations often deviated from 1 and 0 respectively. We hypothesize that our deviations in the slope and intercept are caused by (slight) differences in boundary conditions between the FE model and the experiment. Displacement measures are highly sensitive to the applied boundary conditions^30^; hence, small instabilities in the experimental setup might already affect the displacement validation, e.g. contact at the loading cup not being perfectly frictionless or small motions at the distal fixation. We found only one other study that performed a similar validation of the displacements^20^, yet on a smaller dataset (5 femurs) and for a different region of the femur, i.e. the medial and lateral femoral shaft and superior neck surface as opposed to the anterior surface in this study. Despite the different regions, similar correlation results (0.869 < R^2^ < 0.999) were observed for their validation of the total displacement magnitude. Also, that study found some variation in the slope (0.797 < slope < 1.244), which they attributed to experimental issues such as motion in the mounting jig. In contrast, they found an intercept that was consistently equal to 0, although this was inherent to their methodology by introducing a reference point that calibrated the intercept to 0.

Strain measurements are less sensitive to differences in boundary conditions since they are not affected by rigid body motions. This explains why the range of values of the nominal strains were matching more closely compared those of the displacements. Still, the slope of the regression line was often smaller than 1, indicating that our FE model underestimated the experimental strain values. The correlations had an R^2^ between 0.63 and 0.9 and a NRMSE between 5 and 10%, which is moderate in comparison to previous related studies^20–23^. These moderate results were attributed to several factors. First, the four previous studies used a geometry-based mesh consisting of tetrahedral elements^20–23^, which can reconstruct the contours of the femur with a smooth surface. Instead, we made use of a voxel-based mesh consisting of brick-shaped elements that are directly converted from the voxels in the CT scan. Our rationale for using this meshing approach is that it is robust and fast, can be highly automated and allows to make use of efficient FE solvers dedicated for voxel-based meshes. However, it results in a jagged surface representation and larger partial volume effects in the surface elements and surface strain accuracy is known to be a weak spot for these jagged surface meshes. Second, the strain measurements were affected by the presence of vessel holes, which have already shown previously to deteriorate the validation results^20,23^. They result in strain concentrations in the DIC, likely caused by local damage or by artifacts such as leakage of bone marrow or loosing of the paint, which are not captured by the FE model. Third, we analyzed the nominal strain magnitude along the loading axis as opposed to the principal strain magnitudes in previous studies^20–23^. Hereby, we apply linear regression only to one single parameter, while previous studies have combined both the minimal and maximal principal strains magnitudes in a single regression plot. This pooled analysis can somewhat inflate the correlation coefficient by fitting a regression line between two point clouds that are separated from each other. This makes it difficult to directly compare the resulting correlation coefficients. The reason why we chose for a validation of the nominal strain in contrast to the principal strains was related to our use of a voxel-based mesh. An underlying hypothesis when using the principal strains for validation is that the cortical surface elements in the FE model act as a sort of outer shell, where the maximal and minimal principal strains arise in a plane almost tangential to the surface. Hence, they enable a comparison against DIC, which measures only 2D strains in a plane tangential to the surface. However, the jagged surface representation in a voxel-based mesh might hamper this hypothesis of the principal axes alignment. In contrast, the nominal strain analysis allows an evaluation along a fixed strain axis, which in our opinion provides a more representative validation of the performance of our voxel-based FE model.

Despite the explained inaccuracies regarding the absolute agreement in surface displacements and strains, our model demonstrated a very good qualitative match for the relative distributions (Figs. 4 and 5). This proves the ability of our FE model to accurately simulate the deformation behavior, both before and after cement augmentation. The current implementation of the FE model thereby offers a useful biomechanical tool for analyzing the mechanical effect of femoroplasty. However, for applications where precise measurements of the absolute surface strains are needed, our model might need some refinement. This would require a further investigation to evaluate which are the most important modelling parameters that would need to be adapted to improve the surface strain accuracy.

To evaluate the mechanical effectiveness of femoroplasty, an uncertainty that currently remains is what level of strength improvement is required to prevent post-operative fractures^31,32^. In an experimental setup with artificially created lesions, this effectiveness could be assessed by analyzing whether femoroplasty could restore the initial bone strength before creation of the lesions. Such analysis was performed by Kaneko et al.^8^*,* where they demonstrated that the strength of an augmented metastatic femur reached on average 94.7% (± 8.7%) of the strength of their intact contralateral femur. In our study, we could not draw this conclusion from the experimental results since no intact femurs were mechanically tested to failure. However, we could mimic this analysis numerically by applying our FE model on the CT scans of the intact femurs that were available. Based on these FE results, the augmented strength would be on average 102.6% (± 3.4%) of the intact strength. This result as well as the one by Kaneko et al.^8^ indicate that femoroplasty can effectively restore the intact bone strength, but we should be careful in generalizing this conclusion as both datasets remain relatively small.

There are a number of limitations of our study. First, the artificial lesions do not capture the large variation in shape, dimension and location of real lytic lesions. Moreover, they were created by total bone removal, which assumes them to be fully osteolytic, while they often occur in a mixed form with a blastic component as well. This total bone removal also does not take into account the structure of osteolytic metastatic tissue itself and its interactions with the surrounding bone tissue, which might require a more complex constitutive description^33,34^*.* These aspects might generate some uncertainty regarding the performance of our FE model in real metastatic patients. Second, the material properties assigned to the cement augmented region were based on results under idealized circumstances with almost no cement porosity^26^. In addition, perfect binding was assumed between bone and cement. These aspects might have overestimated the real properties of the cement augmented region. This could be the underlying reason why the FE models resulted in a slightly larger overestimation of the strength and underestimation of the surface strains in the augmented femurs compared to the defect femurs. Third, as mentioned earlier, further investigation on the surface strain accuracy is required when absolute strains measurements need to be recorded.

To conclude, in this study we validated a voxel-based FE model simulating femoroplasty of artificial lytic lesions in terms of global strength and local surface displacements and strains. Despite some inaccuracies in the surface measurements, the FE model accurately predicted the global bone strength and qualitative deformation behavior, both before and after femoroplasty. The developed model can offer a useful biomechanical tool to assist clinicians in assessing the need for prophylactic augmentation in patients with metastatic bone disease, as well as in identifying suitable patients for femoroplasty.

## Supplementary Information

Supplementary Information.

## Data Availability

The datasets generated during and/or analyzed during the current study are available from the corresponding author on reasonable request.

## References

[1] Gendi K, Hennessy D, Heiner J (2016). The burden of metastatic disease of the femur on the medicare system. Springerplus.

[2] Mavrogenis AF (2012). Survival analysis of patients with femoral metastases. J. Surg. Oncol..

[3] Ratasvuori M (2013). Insight opinion to surgically treated metastatic bone disease: Scandinavian Sarcoma Group Skeletal Metastasis Registry report of 1195 operated skeletal metastasis. Surg. Oncol..

[4] Philipp TC, Mikula JD, Doung YC, Gundle KR (2020). Is there an association between prophylactic femur stabilization and survival in patients with metastatic bone disease?. Clin. Orthop. Relat. Res..

[5] Feng H (2016). CT-guided percutaneous femoroplasty (PFP) for the treatment of proximal femoral metastases. Pain Physician.

[6] Deschamps F (2012). Cementoplasty of metastases of the proximal femur: Is it a safe palliative option?. J. Vasc. Interv. Radiol..

[7] Horbach AJ (2020). Biomechanical in vitro examination of a standardized low-volume tubular femoroplasty. Clin. Biomech..

[8] Kaneko TS, Skinner HB, Keyak JH (2007). Feasibility of a percutaneous technique for repairing proximal femora with simulated metastatic lesions. Med. Eng. Phys..

[9] Cazzato RL (2015). Percutaneous long bone cementoplasty for palliation of malignant lesions of the limbs: A systematic review. Cardiovasc. Intervent. Radiol..

[10] Sas A, Tanck E, Sermon A, van Lenthe GH (2020). Finite element models for fracture prevention in patients with metastatic bone disease: A literature review. Bone Rep..

[11] Benca E (2019). QCT-based finite element prediction of pathologic fractures in proximal femora with metastatic lesions. Sci. Rep..

[12] Eggermont F (2020). Patient-specific finite element computer models improve fracture risk assessments in cancer patients with femoral bone metastases compared to clinical guidelines. Bone.

[13] Goodheart JR, Cleary RJ, Damron TA, Mann KA (2015). Simulating activities of daily living with finite element analysis improves fracture prediction for patients with metastatic femoral lesions. J. Orthop. Res..

[14] Sternheim A (2018). Pathological fracture risk assessment in patients with femoral metastases using CT-based finite element methods: A retrospective clinical study. Bone.

[15] Kaneko TS, Skinner HB, Keyak JH (2008). Lytic lesions in the femoral neck: Importance of location and evaluation of a novel minimally invasive repair technique. J. Orthop. Res..

[16] Basafa E (2013). Patient-specific finite element modeling for femoral bone augmentation. Med. Eng. Phys..

[17] Sas A, Van Camp D, Lauwers B, Sermon AA, van Lenthe GHH (2020). Cement augmentation of metastatic lesions in the proximal femur can improve bone strength. J. Mech. Behav. Biomed. Mater..

[18] Grassi L, Isaksson H (2015). Extracting accurate strain measurements in bone mechanics: A critical review of current methods. J. Mech. Behav. Biomed. Mater..

[19] Dickinson AS, Taylor AC, Ozturk H, Browne M (2010). Experimental validation of a finite element model of the proximal femur using digital image correlation and a composite bone model. J. Biomech. Eng..

[20] Katz Y, Yosibash Z (2020). New insights on the proximal femur biomechanics using digital image correlation. J. Biomech..

[21] Helgason B (2014). Development of a balanced experimental-computational approach to understanding the mechanics of proximal femur fractures. Med. Eng. Phys..

[22] Grassi L, Väänänen SP, Ristinmaa M, Jurvelin JS, Isaksson H (2016). How accurately can subject-specific finite element models predict strains and strength of human femora? Investigation using full-field measurements. J. Biomech..

[23] Kok J, Grassi L, Gustafsson A, Isaksson H (2021). Femoral strength and strains in sideways fall: Validation of finite element models against bilateral strain measurements. J. Biomech..

[24] Grassi L (2020). Elucidating failure mechanisms in human femurs during a fall to the side using bilateral digital image correlation. J. Biomech..

[25] Sas A, Ohs N, Tanck E, van Lenthe GH (2020). Nonlinear voxel-based finite element model for strength assessment of healthy and metastatic proximal femurs. Bone Rep..

[26] Sas A, Helgason B, Ferguson SJ, van Lenthe GH (2021). Mechanical and morphological characterization of PMMA/bone composites in human femoral heads. J. Mech. Behav. Biomed. Mater..

[27] Keyak JH, Kaneko TS, Tehranzadeh J, Skinner HB (2005). Predicting proximal femoral strength using structural engineering models. Clin. Orthop. Relat. Res..

[28] Faulkner KG, Glüer CC, Grampp S, Genant HK (1993). Cross-calibration of liquid and solid QCT calibration standards: Corrections to the UCSF normative data. Osteoporos. Int..

[29] Flaig, C. A highly scalable memory efficient multigrid solver for micro-finite element analyses. *PhD Thesis ETH Zurich* (2012). 10.3929/ethz-a-007613965

[30] Gröning F, Liu J, Fagan MJ, O’Higgins P (2009). Validating a voxel-based finite element model of a human mandible using digital speckle pattern interferometry. J. Biomech..

[31] Varga P, Hofmann-Fliri L, Blauth M, Windolf M (2016). Prophylactic augmentation of the osteoporotic proximal femur: Mission impossible?. Bonekey Rep..

[32] Kok J (2019). Fracture strength of the proximal femur injected with a calcium sulfate/hydroxyapatite bone substitute. Clin. Biomech..

[33] Falcinelli C, Di Martino A, Gizzi A, Vairo G, Denaro V (2020). Fracture risk assessment in metastatic femurs: A patient-specific CT-based finite-element approach. Meccanica.

[34] Whyne CM, Hu SS, Workman KL, Lotz JC (2000). Biphasic material properties of lytic bone metastases. Ann. Biomed. Eng..

