# Supplementary material for: Experimental validation of a voxel-based finite element model simulating femoroplasty of lytic lesions in the proximal femur
Source: Sci Rep. 2022 May 9;12:7602. doi: 10.1038/s41598-022-11667-x (PMC9085891; doi:10.1038/s41598-022-11667-x)
Supplement: Supplementary file 1 — Supplementary Information. [file 41598_2022_11667_MOESM1_ESM.pdf]

# Supplementary material

## Experimental validation of a voxel-based finite element model simulating femoroplasty of lytic lesions in the proximal femur

Amelie Sas, An Sermon, G. Harry van Lenthe

**Content:**

S1: Definition of bone material properties

S2: Force-displacement curves

S3: Supplementary figures of the displacement validation

S4: Supplementary figures of the nominal strain validation

# S1: Definition of bone material properties

The nonlinear material properties implemented in the FE models were obtained from Keyak *et al.* [1]. We refer to this reference for more detailed information. The material relationships that were directly required for implementing the properties in the FE model are summarized below (Table S1.1)

***Table S1.1**: Nonlinear material relationships used in the FE models according to the definitions of Keyak et al.* [1]. *For interpretation of the material parameters we refer to Fig S1.1.*

| **Parameter** | **Material relation** | |
|---|---|---|
| Ash density ($g/cm^3$) | $\rho_{ash} = 0.0633 + 0.887\ \rho_{CHA}$ | (trabecular and cortical) |
| Elastic modulus (MPa) | $E = 14900\ \rho_{ash}^{1.86}$ | (trabecular and cortical) |
| Ultimate strength (MPa) | $S = 102\ \rho_{ash}^{1.8}$ | (trabecular and cortical) |
| Plastic strain (-) | $\varepsilon_{AB} = 0.00189 + 0.0241\ \rho_{ash}$ * | (trabecular) |
| | $\varepsilon_{AB} = 0.0184 - 0.0100\ \rho_{ash}$ * | (cortical) |
| Plastic modulus (MPa) | $E_p = -2080\ \rho_{ash}^{1.45}$ * | (trabecular) |
| | $E_p = -1000$ * | (cortical) |
| Minimal stress (MPa) | $\sigma_{min} = 43.1\ \rho_{ash}^{1.81}$ | (trabecular and cortical) |

$\rho_{CHA}$= calcium hydroxyapatite calibrated bone density ($g/cm^3$); $\rho_{ash}$= ash bone density ($g/cm^3$)
* For $\varepsilon_{AB}$ and $E_p$ a correction factor was applied to account for the size of the finite elements as suggested by Keyak *et al.* [1]:

- $\varepsilon_{AB}' = \varepsilon_{AB} \cdot 15/X$ (trabecular)
- $\varepsilon_{AB}' = \varepsilon_{AB} \cdot 5/X$ (cortical)
- $E_p' = (X\ E\ E_p)/(15E - (15 - X)E_p)$ (trabecular)
- $E_p' = (X\ E\ E_p)/(5E - (5 - X)E_p)$ (cortical)

with $X$ equal to the minimal value of 3 (see [2] for more information)

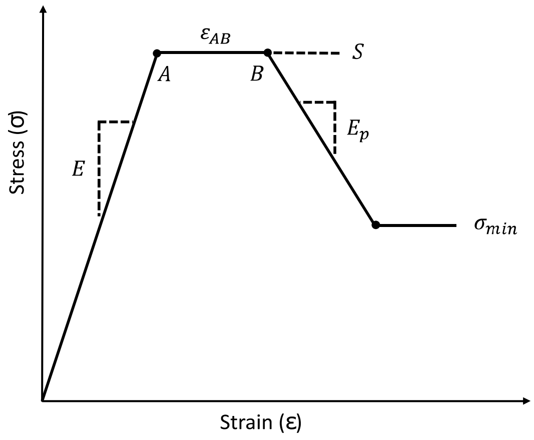


***Fig S1.1**: Nonlinear stress-strain curve with the corresponding material parameters as defined by Keyak et al.* [1]

## References


[1] J.H. Keyak, T.S. Kaneko, J. Tehranzadeh, H.B. Skinner, Predicting proximal femoral strength using structural engineering models, Clin. Orthop. Relat. Res. (2005) 219–228.

[2] J.H. Keyak, I.Y. Lee, D.S. Nath, H.B. Skinner, Postfailure compressive behavior of tibial trabecular bone in three anatomic directions, J. Biomed. Mater. Res. 31 (1996) 373–378.

# S2: Force-displacement curves

Figures comparing the experimental and FE force-displacement curve for all specimen pairs

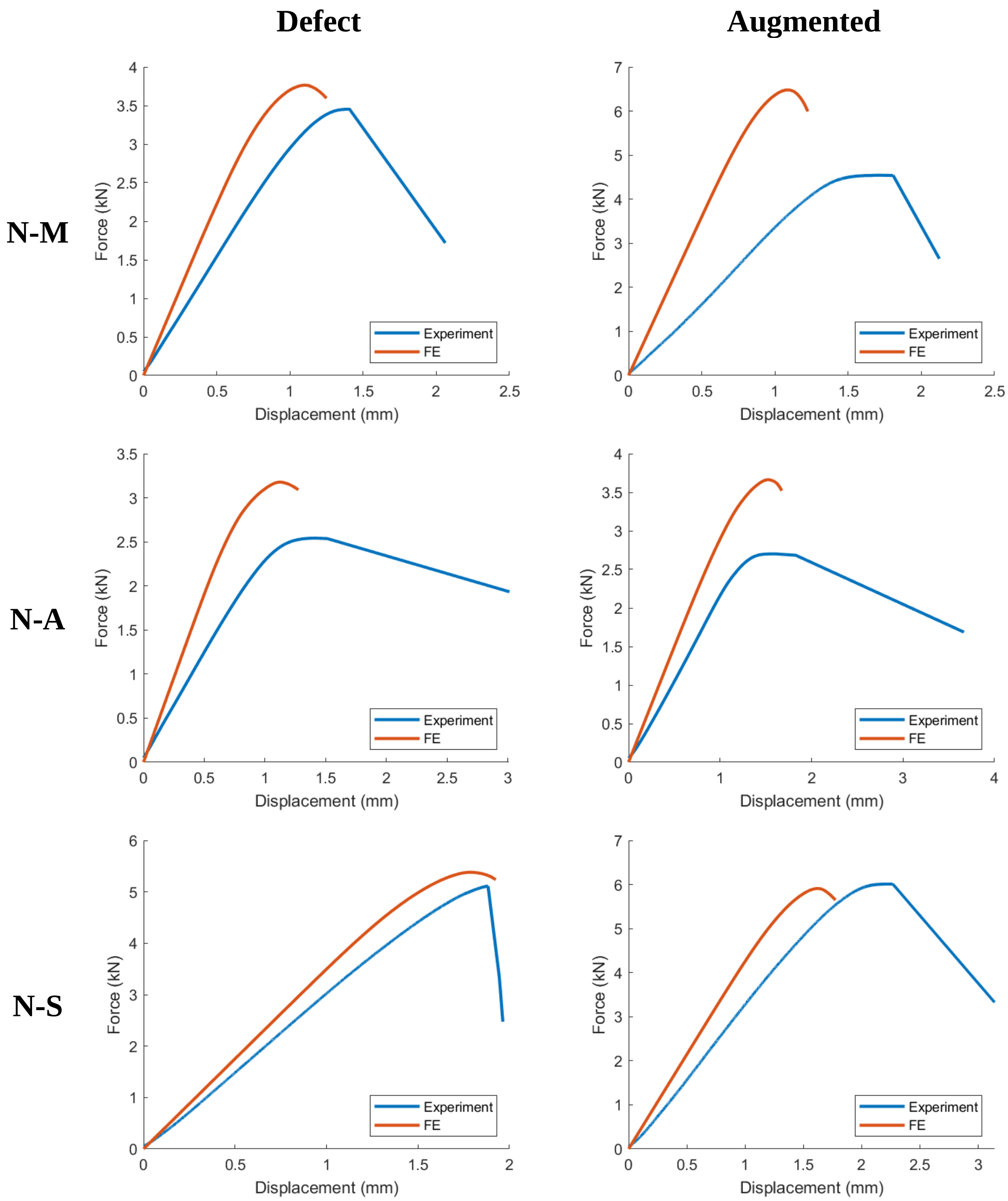

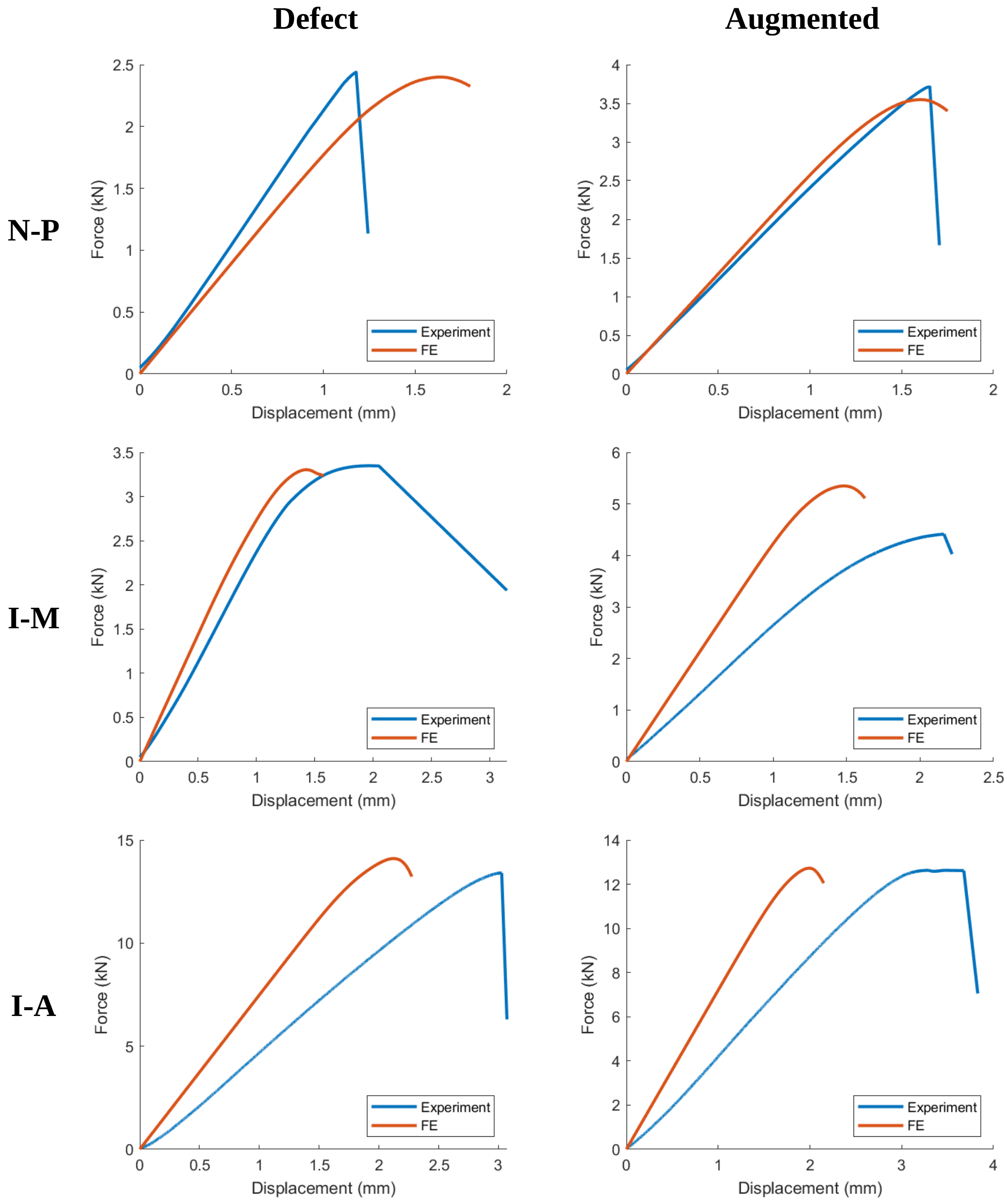
Defect
Augmented
N-P
I-M
I-A
Force (kN)
Displacement (mm)
Experiment
FE
0
0.5
1
1.5
2
2.5
3
3.5
4
5
6
10
15
8
12
14

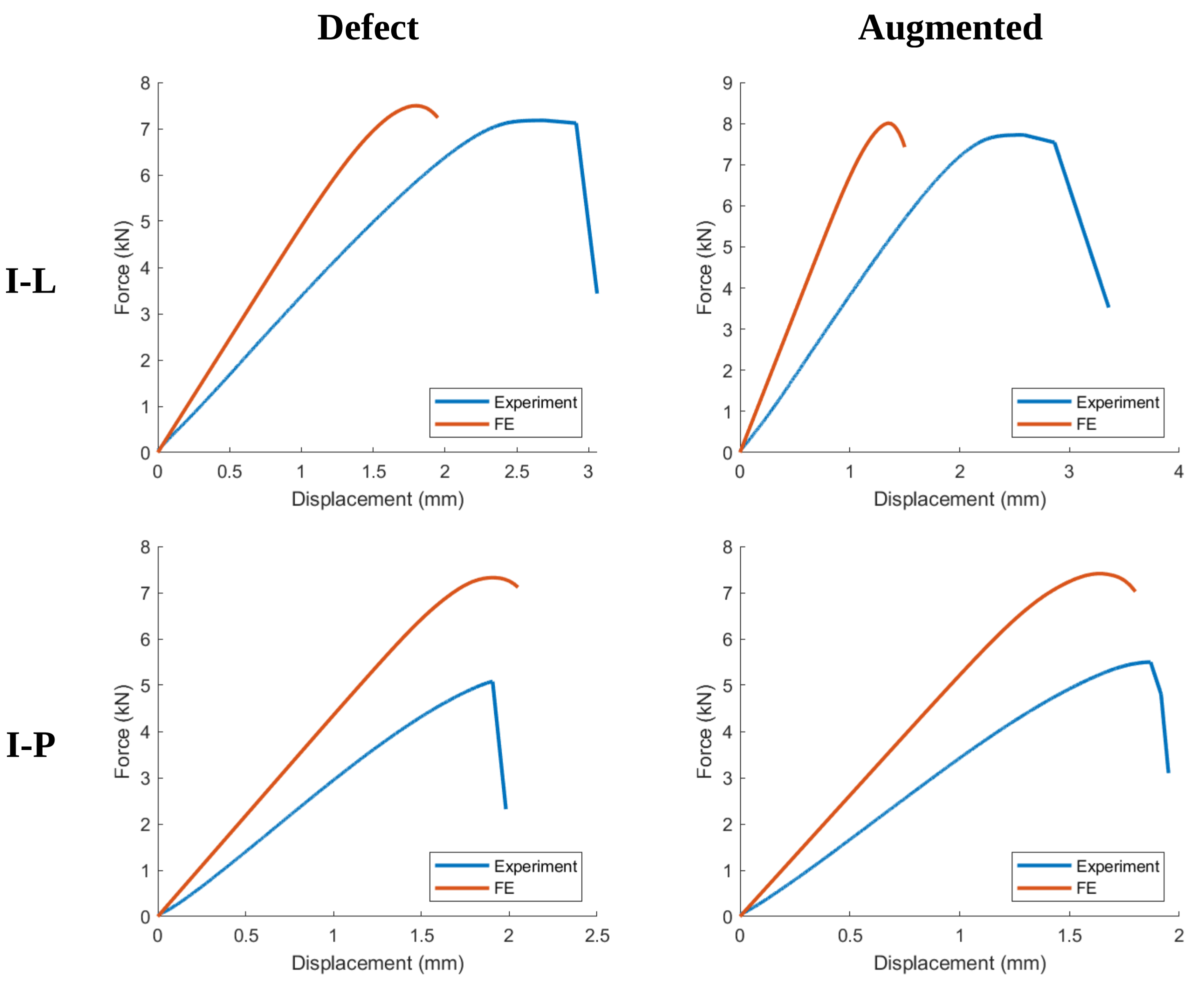
Defect
Augmented
I-L
I-P
Force (kN)
Displacement (mm)
Experiment
FE

# S3: Supplementary figures of the displacement validation

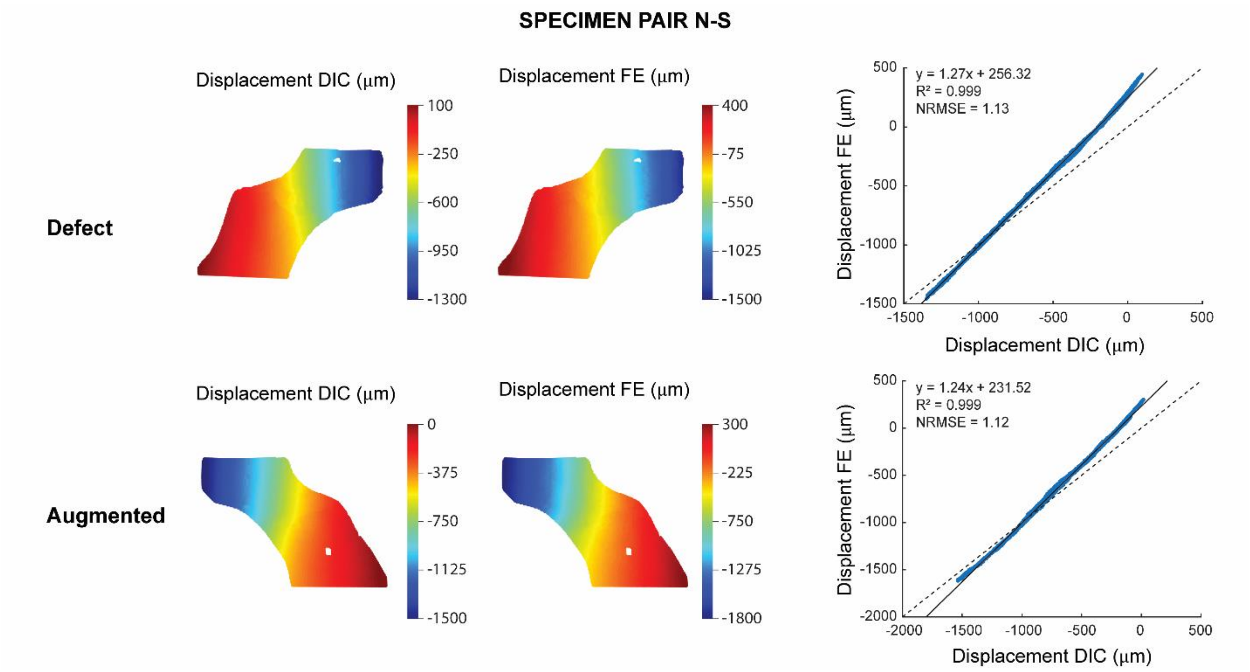


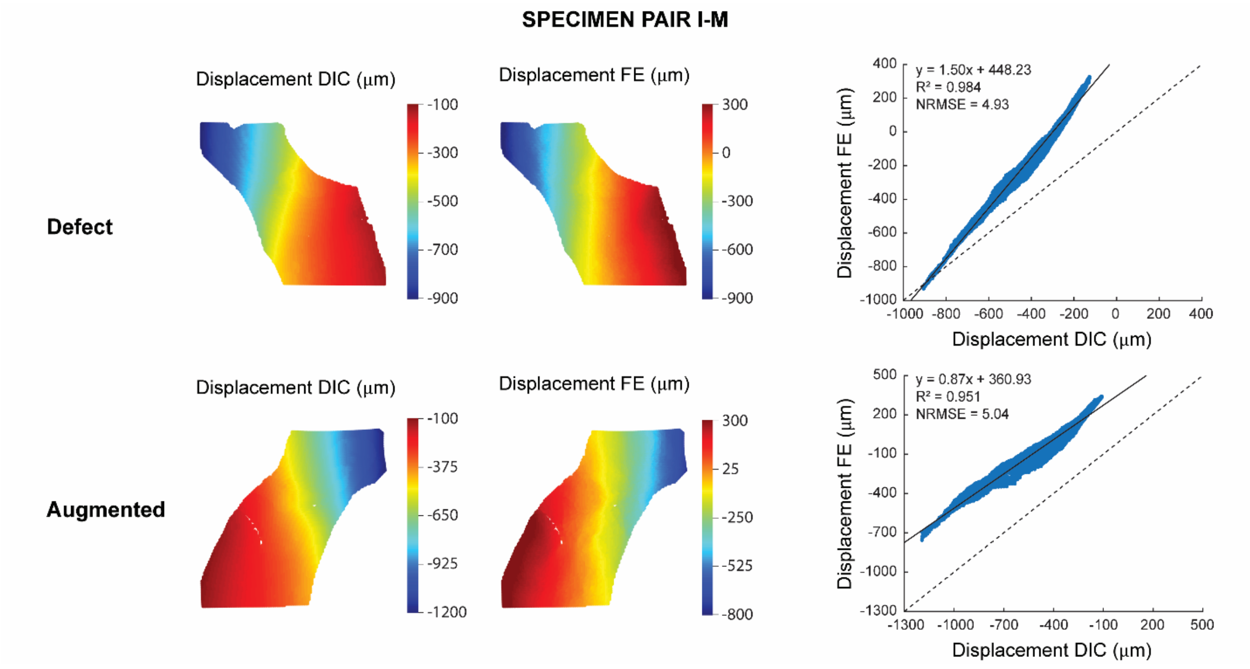

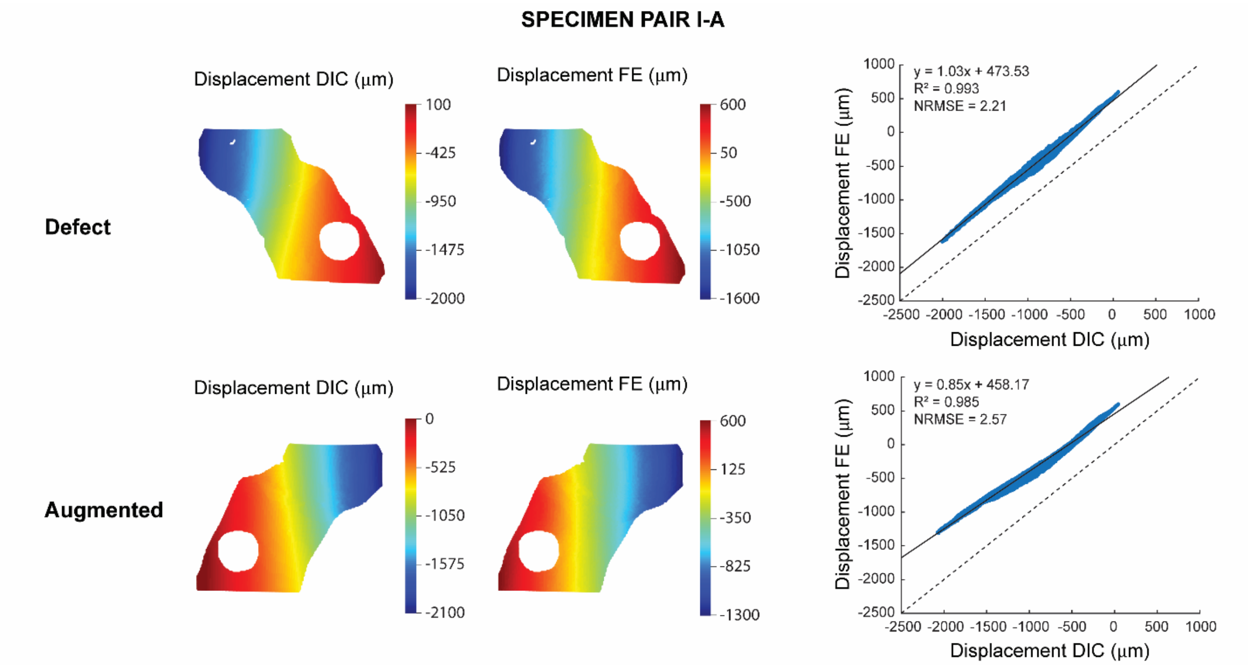
SPECIMEN PAIR I-A
Displacement DIC (μm)
Displacement FE (μm)
Defect
100
-425
-950
-1475
-2000
600
50
-500
-1050
-1600
y = 1.03x + 473.53
R² = 0.993
NRMSE = 2.21
Displacement FE (μm)
Displacement DIC (μm)
Augmented
0
-525
-1050
-1575
-2100
600
125
-350
-825
-1300
y = 0.85x + 458.17
R² = 0.985
NRMSE = 2.57

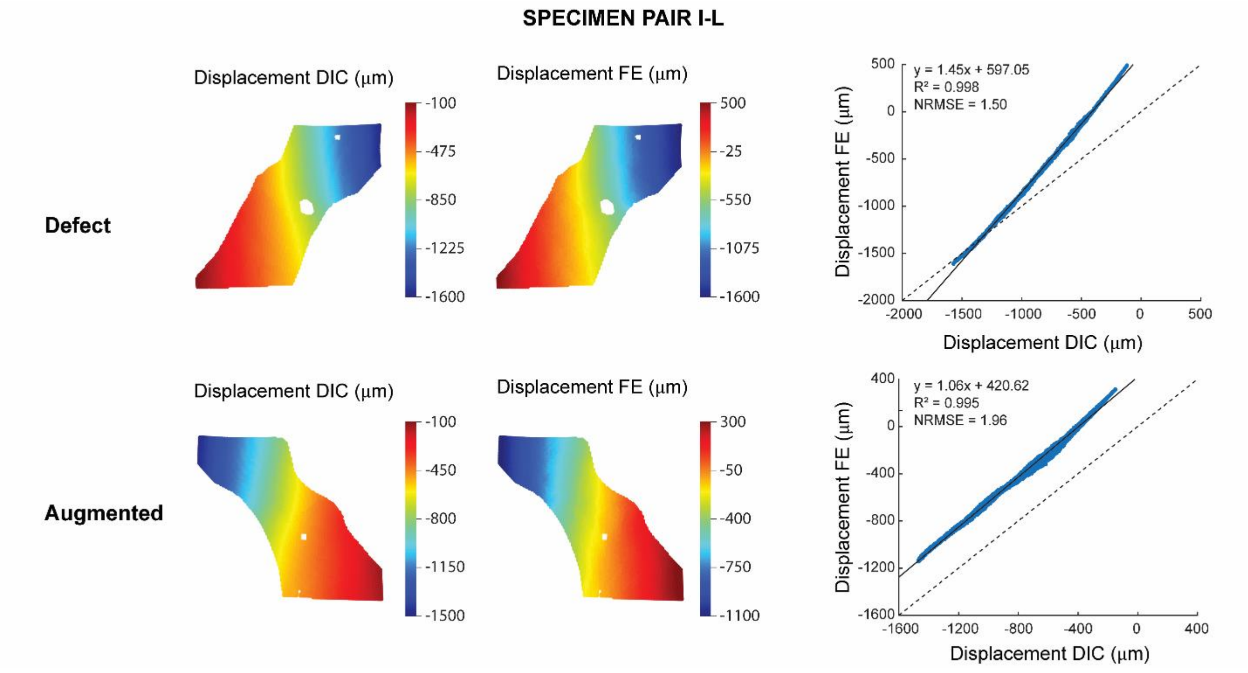
SPECIMEN PAIR I-L
Displacement DIC (μm)
Displacement FE (μm)
Defect
-100
-475
-850
-1225
-1600
500
-25
-550
-1075
-1600
y = 1.45x + 597.05
R² = 0.998
NRMSE = 1.50
Displacement FE (μm)
Displacement DIC (μm)
Augmented
-100
-450
-800
-1150
-1500
300
-50
-400
-750
-1100
y = 1.06x + 420.62
R² = 0.995
NRMSE = 1.96

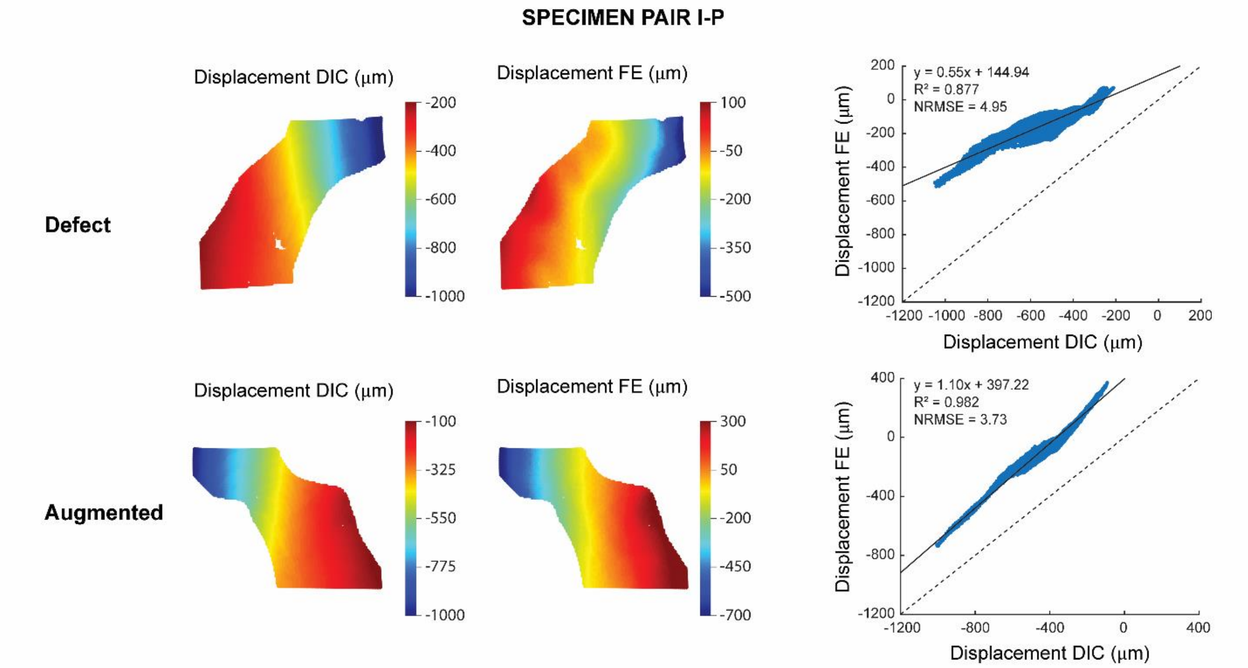

SPECIMEN PAIR I-P
Defect
Augmented
Displacement DIC (μm)
Displacement FE (μm)
y = 0.55x + 144.94
R² = 0.877
NRMSE = 4.95
y = 1.10x + 397.22
R² = 0.982
NRMSE = 3.73

# S4: Supplementary figures of the nominal strain validation

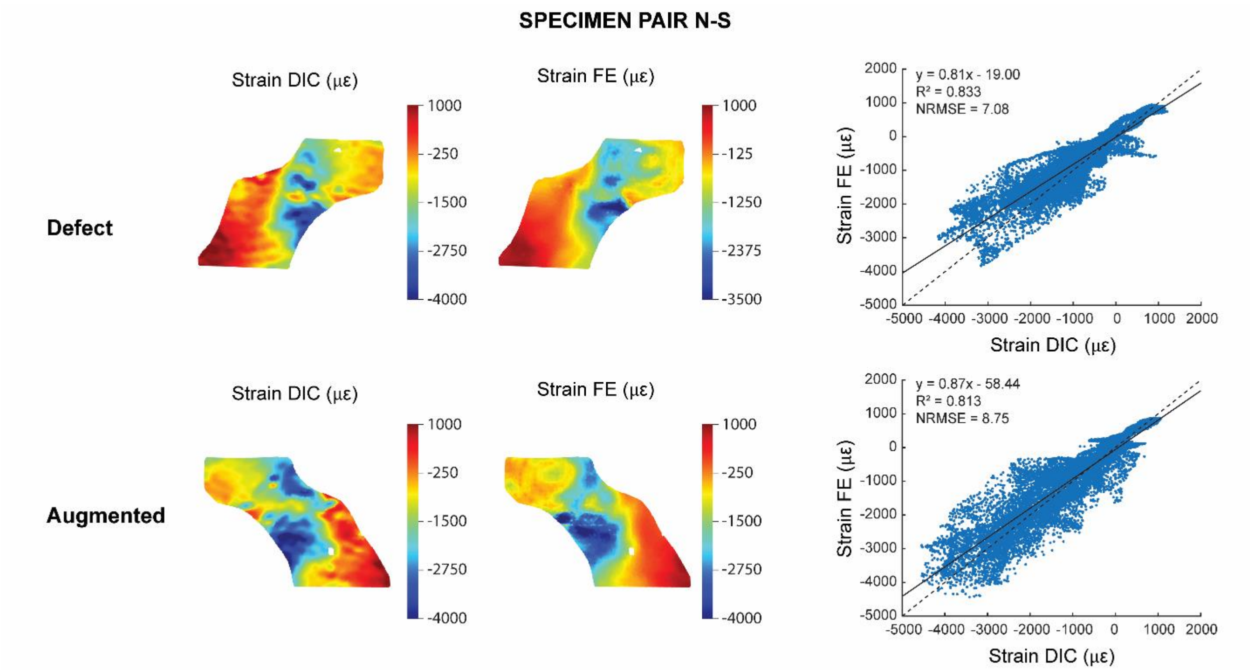


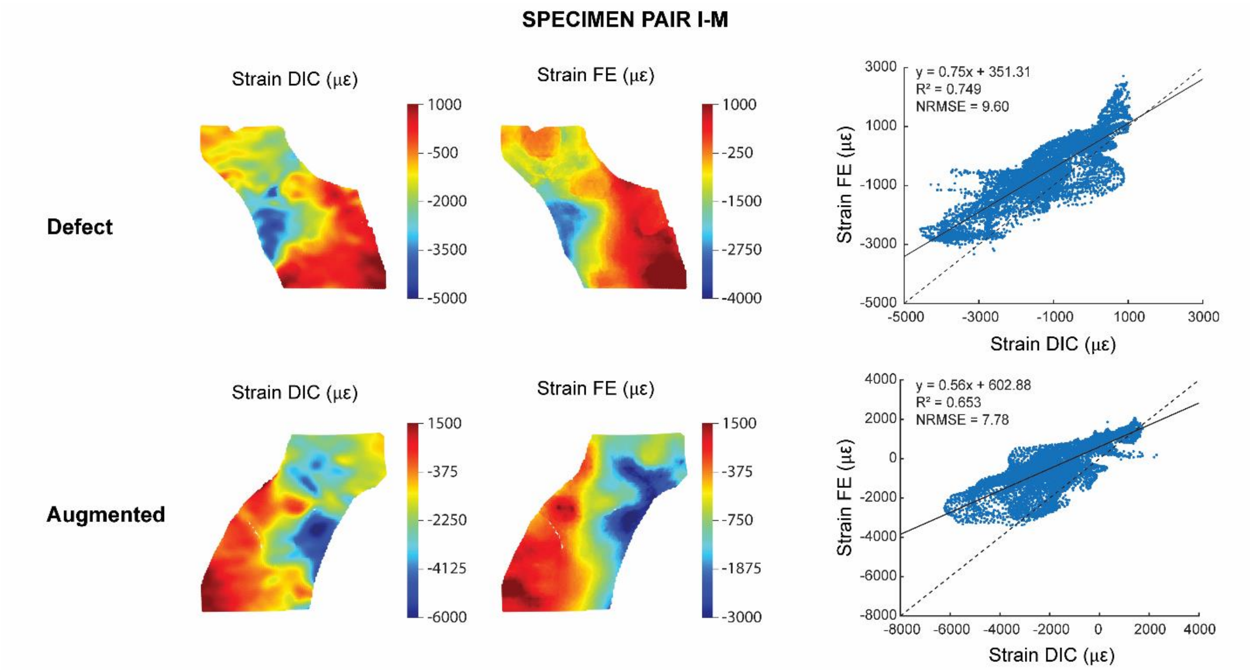

**SPECIMEN PAIR I-A**

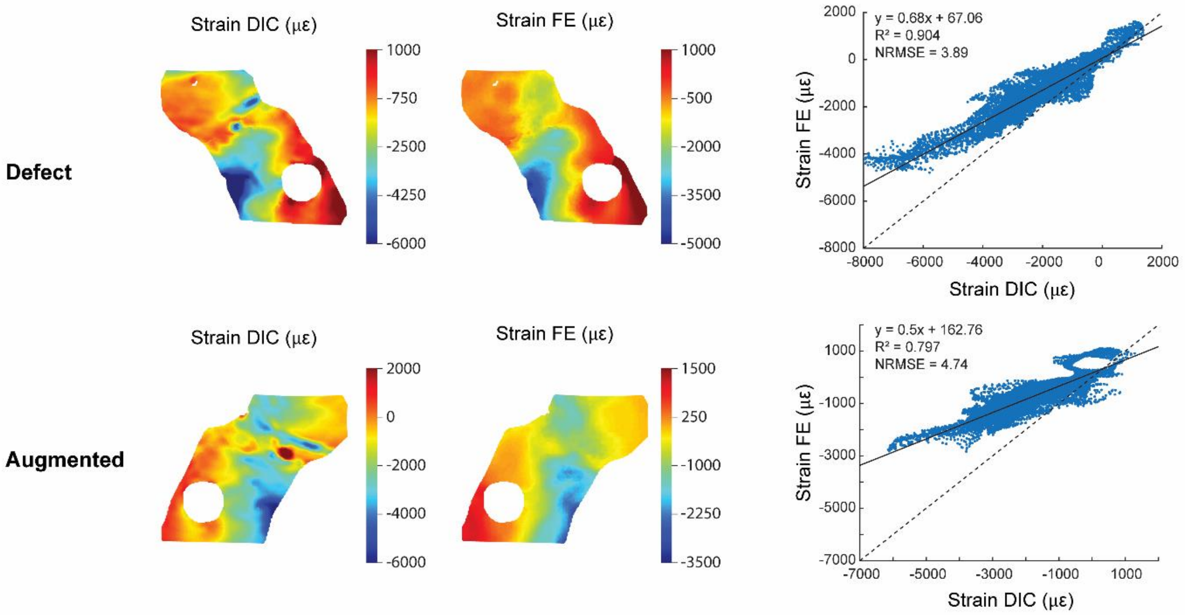


**SPECIMEN PAIR I-L**

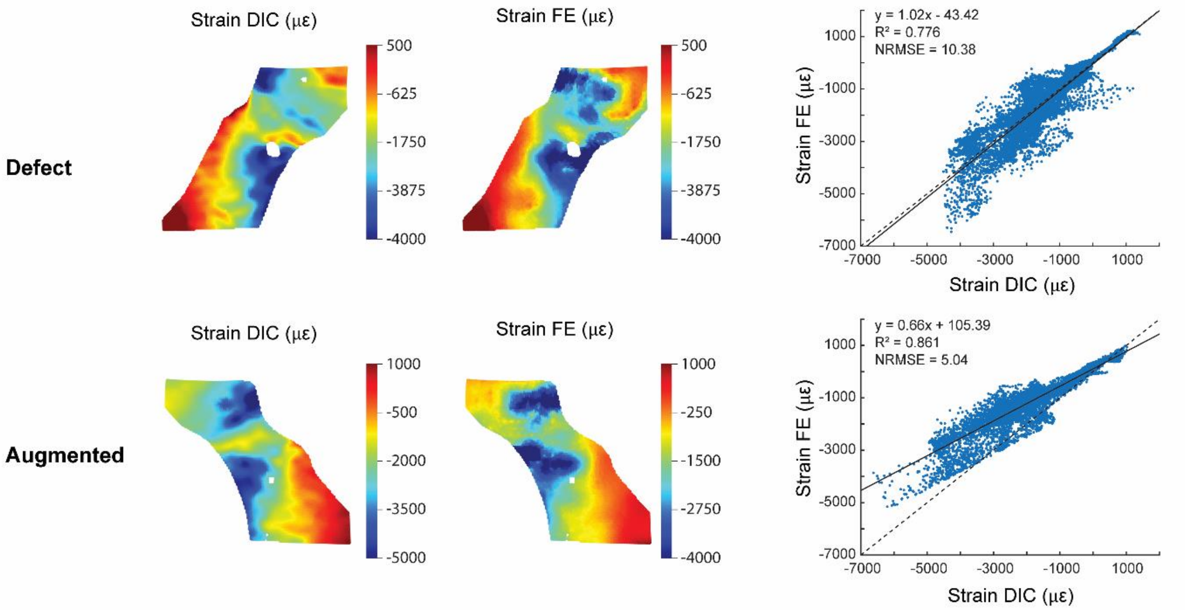

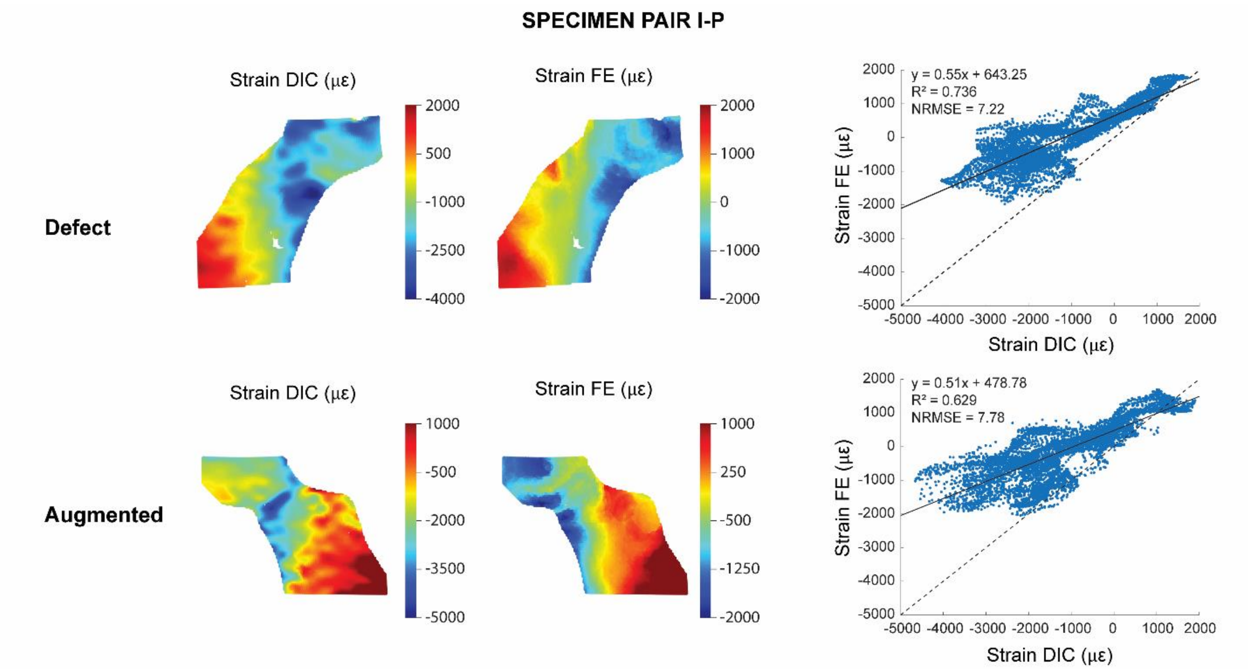
SPECIMEN PAIR I-P
Strain DIC (με)
Strain FE (με)
Defect
Augmented
2000
500
-1000
-2500
-4000
2000
1000
0
-1000
-2000
1000
-500
-2000
-3500
-5000
1000
250
-500
-1250
-2000
y = 0.55x + 643.25
R² = 0.736
NRMSE = 7.22
y = 0.51x + 478.78
R² = 0.629
NRMSE = 7.78
Strain FE (με)
Strain DIC (με)
-5000 -4000 -3000 -2000 -1000 0 1000 2000
